# Stroboscopic Vision When Interacting With Multiple Moving Objects: Perturbation Is Not the Same as Elimination

**DOI:** 10.3389/fpsyg.2018.01290

**Published:** 2018-07-25

**Authors:** Simon J. Bennett, Spencer J. Hayes, Makoto Uji

**Affiliations:** ^1^Research Institute for Sport and Exercise Sciences, Liverpool John Moores University, Liverpool, United Kingdom; ^2^School of Psychology and Neuroscience, University of St. Andrews, St. Andrews, United Kingdom

**Keywords:** stroboscopic vision, MOT, MOA, perceptual-motor, motion, form

## Abstract

Motivated by recent findings of improved perceptual processing and perceptual-motor skill following stroboscopic vision training, the current study examined the performance and acquisition effects of stroboscopic vision methods that afford a different visual experience. In Experiment 1, we conducted a within-subject design study to examine performance of a multiple object tracking (MOT) task in different stroboscopic vision conditions (Nike Vapor Strobe^®^, PLATO visual occlusion, and intermittent display presentation) operating at 5.6, 3.2, or 1.8 Hz. We found that participants maintained MOT performance in the Vapor Strobe condition irrespective of strobe rate. However, MOT performance deteriorated as strobe rate was reduced in the other two stroboscopic vision conditions. Moreover, at the lowest strobe rate (1.8 Hz) there was an increase in probe reaction time, thus indicating an increased attentional demand due to the stroboscopic vision. In Experiment 2, we conducted a mixed design study to examine if practice in different stroboscopic vision conditions (Nike Vapor Strobe^®^ and PLATO visual occlusion) influenced acquisition of a novel precision-aiming task [i.e., multiple object avoidance (MOA) task] compared to a normal vision group. Participants in the PLATO visual occlusion group exhibited worse performance during practice than the Vapor Strobe and normal vision groups. At post-test, the Vapor Strobe group demonstrated greater success and reduced end-point error than the normal vision and PLATO groups. We interpret these findings as showing that both an intermittent perturbation (Nike Vapor Strobe^®^) and elimination (PLATO visual occlusion and intermittent display presentation) of visual motion and form are more attention demanding (Experiment 1), however, the intermittent perturbation, but not elimination, of visual motion and form can facilitate acquisition of perceptual-motor skill (Experiment 2) in situations where it is necessary to maintain and update a spatio-temporal representation of multiple moving objects.

## Introduction

There is no doubting the importance of vision in guiding behavior as we interact within our surrounds, whether it is for object manipulation during tool use, or ambulatory activities such as descending a staircase or navigating along a busy road. However, it is not so obvious that many activities are supported by an interrupted flow of visual information, such as when making a saccade to shift overt attention (i.e., saccadic masking) or tracking an object that is intermittently occluded by other objects or surfaces. Fortunately, the human brain has developed predictive processes that help fill in the gaps in missing visual information (for a review see Bosco et al., 2015), thus resulting in the conscious experience of perceptual stability and constancy. That said, the ability to maintain accurate behavior in such situations is not infallible. For everyday tasks such as reaching and grasping, precision stepping, or one-handed catching, successful performance requires brief visual samples to be separated by no longer than 80–150 ms (Elliott et al., 1994a,b).

Recently, investigators have begun to consider whether practice under such conditions (i.e., stroboscopic vision) can facilitate the development of perceptual and perceptual-motor skill. Analogous to altitude training for the endurance athlete (Appelbaum and Erickson, 2016), the basic premise is that practicing in stroboscopic vision encourages improved visual-cognitive processing in order to adapt to the suboptimal information available during intermittent periods of occlusion. Processes shown to transfer positively when vision is subsequently restored to normal include short-term visual memory (Appelbaum et al., 2012), coincidence-anticipation timing (Smith and Mitroff, 2012), and motion coherence and attention in central vision (Appelbaum et al., 2011). Adaptation in such underlying processes following stroboscopic vision training has been implicated in improvements in sports-specific skills in ice-hockey (Mitroff et al., 2013) and baseball (Clark et al., 2012), thereby providing some support for anecdotal reports of stroboscopic vision training by elite athletes in sports including American football, basketball and alpine skiing.

While the potential impact of general stroboscopic vision training on acquisition of a broad range of perceptual and perceptual-motor skill looks promising, there are several issues that remain to be considered. Of particular interest to the current study is the impact of the visual experience afforded by different methods of creating stroboscopic vision. In the earlier work that examined the impact of stroboscopic vision on performance of perceptual (Scholl and Pylyshyn, 1999; Keane and Pylyshyn, 2006) or perceptual-motor tasks (Elliott et al., 1994a,b), vision of the imperative stimulus was intermittently eliminated. For example, Elliott et al. (1994a; 1994b) used PLATO visual occlusion eyewear with liquid crystal lenses (Translucent Technologies Inc.) that change rapidly between open and closed states (Milgram, 1987). The lenses are transparent in the open state and are similar to looking through clear glass. There is equivalent light transmission in the closed state when the lenses are translucent with a “milky” appearance, but the light is scattered. This prevents image formation on the retina and the perception of motion and form. This contrasts to the eyewear (i.e., Nike Vapor Strobe^®^) used in more recent work by Appelbaum, Mitroff and colleagues, which have lenses that switch between more or less transparent states. In the latter state, the lenses operate as neutral density filters, thereby reducing light transmission (for more detail see methods). Although not experimentally verified with the Nike Vapor Strobe eyewear, or other strobe eyewear that are currently commercially available (Senaptec Strobe, Visionup, VIMA Rev Sport), it is well known that low light conditions impact upon visual acuity (von Noorden and Burian, 1959), contrast sensitivity (Owsley et al., 1983), and ocular accommodation (Johnson, 1976). These basic visual functions are important for higher level perception of object motion and form (for a review see Burton et al., 2016), and thus their perturbation could potentially explain why performance of perceptual-motor tasks is more effortful and attentionally demanding in conditions of stroboscopic vision (Ballester et al., 2017). This interpretation would also add credence to anecdotal reports that participants exhibit more focused attention on an approaching object when practicing catching tasks in stroboscopic vision (Athletic Republic, 2011).

In the current study, we first compared the effect of different stroboscopic vision methods on performance of multiple object tracking (MOT), and subsequently the acquisition of a novel but related precision-aiming task, more commonly known as multiple object avoidance (MOA) (Mackenzie and Harris, 2017). MOT requires participants to track and disambiguate multiple objects from distractors (Scholl and Pylyshyn, 1999; Alvarez et al., 2005), whereas MOA requires a cursor to be moved from a home position to an end target while avoiding multiple moving objects. Therefore, both MOT and MOA demand distributed and sustained visual attention in order to maintain a persistent spatial representation of the moving surrounds (Pylyshyn and Storm, 1988; Cavanagh and Alvarez, 2005; Fehd and Seiffert, 2008; Ericson and Christensen, 2012; Mackenzie and Harris, 2017). As well as providing important experimental control, these tasks are relevant because they have perceptual processes in common with situations faced in everyday settings. For instance, perceptual training on a lab-based MOT task has recently been shown to convey positive transfer to on-field performance of essential soccer skills (Romeas et al., 2016), whereas performance of MOA predicts driving behavior (Mackenzie and Harris, 2017). Understanding whether and how different stroboscopic vision methods affect performance during lab-based tasks (perceptual and perceptual-motor) requiring MOT is therefore an important step on the way to designing protocols that could facilitate the development of perceptual-motor processes that transfer to real-world settings.

## Experiment 1 – Multiple Object Tracking Task

### Methods

#### Participants

Eighteen young adults (*M* = 21.8 years of age, *SD* = 1.8) volunteered to take part in the study. All participants had normal or corrected-to-normal vision. Participants were provided with general information about the task and stimulus prior to giving informed written consent. The study was reviewed and approved by the research ethics committee of the Research Institute for Sport and Exercise Sciences at Liverpool John Moores University. All procedures were conducted in accordance with the ethical guidelines of Liverpool John Moores University and the 1964 Declaration of Helsinki.

#### Apparatus and Task

A 22-inch CRT computer monitor (Iiyama MA203DT Vision Master 513, Tokyo, Japan) operating with a resolution of 1280 × 1024 pixels and a refresh rate of 85 Hz, was connected to a host computer (HP Compaq 8000 Elite, California, United States) running Windows XP operating system. The monitor was placed on a desk at a height of 1.0 m, and at a distance of 0.9 m from the participant, who was sat on a height-adjustable chair.

Participants completed a MOT task (Pylyshyn and Storm, 1988; Cavanagh and Alvarez, 2005), which was realized using the cogent toolbox implemented in MATLAB (The Mathworks, Inc., MA, United States). The main aim of the task was to track four target objects (1.8°visual angle) moving within a group of 12 identical objects (i.e., eight distractors) over a duration of 10 s. The target and distractor objects moved against a black background and within a white rectangular frame that subtended a horizontal and vertical extent of 25.7° and 19.4° respectively. In each trial, the 12 objects moved around the screen in accord with eight pre-programmed linear trajectories. These were randomly selected and formed using object speeds of 8.9, 8.6, 5.5, and 5.0°/s. Object speed was constant within a trial, and when an object reached the surrounding frame, it rebounded with an angle that was equal to the angle of incidence. Objects did not rebound upon collision with each other and instead continued along their trajectory without any change.

**FIGURE 1:**
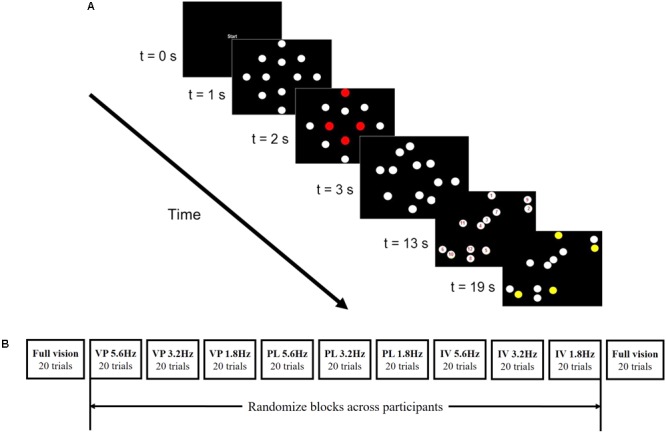
Timeline representation of the MOT task (see text for details) is shown in panel **(A)**. Panel **(B)** shows an example schematic of the MOT task procedure (see text for details).

**Figure 1A** shows a timeline graphical representation of the various stages of a trial on the MOT task. At the beginning of each trial, the word “start” appeared for one second. Then, a static image of the initial positions of 12 white objects was presented for one second. Following the static image, four of the objects were highlighted in red as the targets. After one second, all 12 objects were again drawn in white, and then started to follow the pre-programmed linear trajectories. After 10 s, the 12 white objects stopped moving and were shown stationary in their final position with a number from 1–12 drawn at their center. They remained in this position until the participant verbally indicated the numbers of the four objects they believed to the targets, and the experimenter had pressed the corresponding function keys (F1–F12) on the computer keyboard. The targets were then highlighted in yellow for 1 s in order to provide feedback on the participant’s response. A blank screen then appeared for 1 s, after which the next trial began.

The MOT task was performed in a normal vision condition (i.e., no occlusion) before and after completing the task in three different conditions of stroboscopic vision. In two such conditions (i.e., Vapor Strobe and PLATO) participants wore eyewear with liquid crystal lenses that cycled between “open” and “closed” states. Nike Vapor Strobe^®^ eyewear has a fixed “open” duration of 100 ms and a “closed” duration that can be varied between eight different levels. Here, we used level two, four, and six, which we confirmed using high-speed video equated to “closed” durations of 85, 210, and 460 ms (5.6, 3.2, and 1.8 Hz) respectively. The state of the PLATO eyewear lenses (Translucent Technologies Inc., Toronto, ON, Canada) was controlled using TTL signals from the parallel port of the host computer and was matched to the “open” (100 ms) and “closed” duration of 80, 210, and 460 ms (5.6, 3.2, and 1.8 Hz) of the Nike Vapor Strobe^®^ eyewear. Transmission of ambient room light through the lenses of the eyewear in the “closed” state was measured using a digital light meter (Lutron LX-1108, Taipei, Taiwan). With the meter located directly behind the lens, and placed at 196 cm from a light source (i.e., office lighting), the illuminance was 87 lux for the Nike Vapor Strobe^®^ eyewear and 260 lux for the PLATO visual occlusion eyewear. Without the eyewear lens placed in front of the light meter, the illuminance was 467 lux. For reference, an illuminance of 100 lux is similar to that of a “very dark overcast day” (Schlyter, 2015), while 320 lux is the minimum illuminance for office lighting recommended by the United States Department of Labor. In the third condition, stroboscopic vision of the experimental task was created by intermittently removing the stimuli from the monitor. Accordingly, when there was no visual input regarding the multiple moving objects (i.e., blank screen), the participant could still see the screen edges and surrounds. Given the constraints of the monitor refresh rate, the stimuli in each cycle were drawn for 8 consecutive frames (i.e., 94 ms) and then replaced by a blank screen for seven (82 ms), 18 (212 ms), or 39 (459 ms) frames. This produced strobe frequencies of 5.6, 3.2, and 1.8 Hz, respectively.

In order to quantify attentional demand during the MOT task, probe reaction time was randomly assessed during each block of 20 trials with a 1:4 (probe/no probe) ratio. Participants were required to respond as quickly as possible to an auditory tone (750 Hz for 250 ms) by pressing the left button on a computer mouse (Logitech GX), which was polled via the computer USB port at 1000 Hz. The trial with an auditory tone was randomly determined for each block, whereas the presentation time of the auditory tone within the trial was randomly determined between 4 and 8 s after the 12 objects began to move.

#### Procedure

Before the start of the experiment, participants received an illustration of the screen layout (i.e., 12 white objects and rectangular frame) and pre-scripted instructions regarding the aim of the task, the respective method used to create stroboscopic vision, and strobe rates. They were instructed to track four target objects for 10 s, and to respond to the auditory tone as quickly as possible. They were unaware of the number of object movement patterns, and the number and location of auditory tones. MOT was first completed in a normal visual condition by all participants in order to ensure task familiarization. Participants then completed nine blocks of 20 MOT trials (i.e., one block for each unique combination of three visual condition × three strobe rate). The order of the blocks was completely randomized across participants (see **Figure 1B**). Participants were provided with the opportunity to have a break after every block if they deemed necessary. Finally, MOT was completed a second time in a normal vision condition. This was done in order to assess whether any differences between stroboscopic vision conditions could be explained by a general learning effect.

#### Data Analysis

As a measure of performance on the primary task (MOT), we calculated the arcsine percentage of successful responses. There were 80 potential successful responses (4 successful responses per trial × 20 trials) per block. To examine attentional demand during MOT, mean probe reaction time (ms) was calculated from the difference between issuing the auditory tone and recording the mouse button press. These dependent measures were submitted to separate three visual conditions (Vapor Strobe, PLATO, and intermittent display presentation) × three strobe rate (5.6, 3.2, and 1.8 Hz) repeated designed ANOVA. In the event of a significant main or interaction effect, the Holm-Bonferroni method was used to adjust the *p*-value to maintain a familywise error rate of α = 0.05. For the interaction effects, we sequentially compared strobe rate (i.e., 5.6 vs. 3.2; 3.2 vs. 1.8) for each level of visual condition to give a total of six pairwise comparisons. The same dependent variables were extracted for the normal visual condition, completed pre- and post-stroboscopic vision conditions, and submitted to separate dependent *T*-tests.

### Results

#### Performance

**FIGURE 2:**
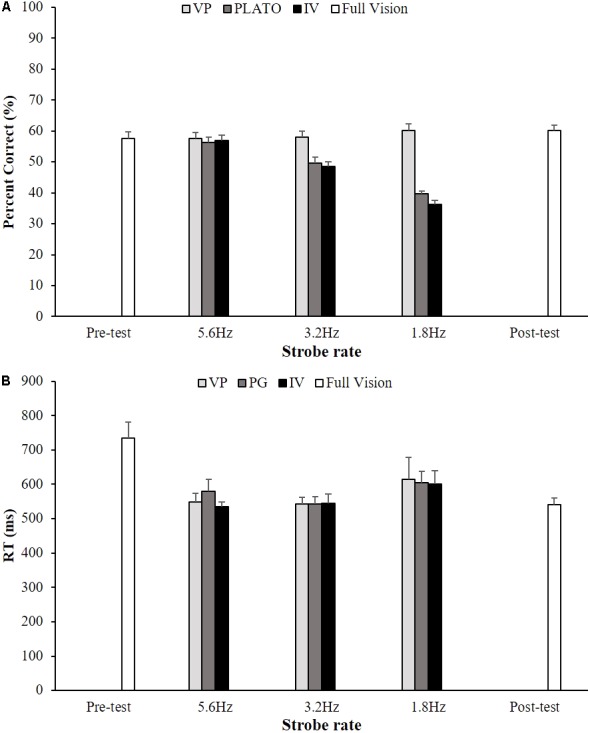
Group mean (+SEM) percent correct responses **(A)** and reaction time **(B)** as a function of stroboscopic vision condition (VP = Nike Vapor Strobe^®^; PL = PLATO Visual Occlusion; IV = Intermittent Visual Display) and strobe rate (5.6, 3.2, and 1.8 Hz). Data from the full vision condition at the pre-test and post-test is included for comparison.

For arcsine percentage of successful trials there was a significant main effect of visual condition, *F*(2,34) = 56.9, *p* < 0.01, $\eta_{p}^{2}$ = 0.77, strobe rate, *F*(2,34) = 89.2, *p* < 0.01, $\eta_{p}^{2}$ = 0.84, and an interaction between visual condition and strobe rate, *F*(4,68) = 28.5, *p* < 0.01, $\eta_{p}^{2}$ = 0.63 (**Figure 2A**). In the Vapor Strobe condition performance was maintained irrespective of strobe rate. However, for the other two strobe vision conditions there was a significant reduction in performance for each consecutive reduction in strobe rate. For probe reaction time there was a significant main effect of strobe rate, *F*(2,34) = 4.1, *p* < 0.05, $\eta_{p}^{2}$ = 0.20 (**Figure 2B**), whereas there was no significant main effect of visual condition, *F*(2,34) = 0.3, *p* > 0.05, $\eta_{p}^{2}$ = 0.02, and interaction between visual condition and strobe rate, *F*(4,68) = 0.3, *p* > 0.05, $\eta_{p}^{2}$ = 0.02. Probe reaction time was significantly longer for a strobe rate of 1.8 Hz (607 ms) compared to 3.2 Hz (541 ms). Probe reaction time for a strobe rate of 5.6 Hz was 554 ms.

#### Adaptation

There was no change in percentage of successful trials between the pre-test and post-test in a normal vision condition, *F*(1,17) = 1.53, *p* > 0.05, $\eta_{p}^{2}$ = 0.08 (see **Figure 2A**). There was, however, a significant reduction in probe reaction time from 734 ms to 541 ms, *F*(1,17) = 14.2, *p* < 0.01, $\eta_{p}^{2}$ = 0.45. Additional dependent *T*-tests comparing probe RT in post-test to the nine stroboscopic vision conditions revealed no differences (*p* > 0.05) (see **Figure 2B**).

### Discussion

The current study examined performance on an MOT task in different conditions of stroboscopic vision. Attentional demand of performing MOT in the different conditions was measured at random time points using an auditory probe reaction task. We found that participants exhibited a similarly high percentage of successful trials in the Vapor Strobe conditions irrespective of strobe rate. In contrast, performance deteriorated significantly as strobe rate was reduced in the PLATO condition or when viewing an intermittent display presentation. There was no such interaction effect for probe reaction time, which was significantly longer for the lowest strobe rate irrespective of stroboscopic vision condition.

The findings of this study are consistent with participants allocating more attentional resource to the primary MOT task when faced with the lowest strobe rate (Flombaum et al., 2008). The allocation of greater attentional resource to the primary MOT task seemingly enabled participants to maintain performance in the Vapor Strobe condition, where the low transparency of the lenses in the “closed” state limits transmission of structured light and thus likely perturbs basic visual function that contributes to perception of motion and form. Indeed, had the “closed” state of the Vapor Strobe lenses simply eliminated perception of this visual information, it could be expected that there would be no difference compared to the other stroboscopic vision conditions. In these conditions, increased attentional allocation (i.e., increased probe RT) did not enable participants to maintain performance when vision of the moving cursor and objects was eliminated for more than 200 ms during the “closed” state (<3.2 Hz). Being able to see the surrounds and screen edges in the intermittent display condition did not seem to convey any advantage. A similar deterioration in MOT performance when vision was eliminated for more than 295 ms was reported by Alvarez et al. (2005). In the context of the processes involved in MOT, the deterioration in performance occurs when participants are no longer able to maintain and update the spatio-temporal representation of multiple moving objects between intermittent visual samples.

Additionally, we found that while there was no change in percentage of successful trials between the pre-test and post-test in a normal vision condition, there was a significant reduction in probe reaction time. Dependent *T*-tests comparing probe RT in post-test to the nine stroboscopic vision conditions revealed no differences. These findings indicate that the longer probe RT at pre-test was likely a result of initial familiarization with the MOT task procedure, and thus any differences between stroboscopic vision conditions would not be explained by a general learning effect.

## Experiment 2 – Multiple Object Avoidance Task

Having shown that MOT was influenced by stroboscopic vision condition, a second experiment was designed to examine the acquisition of a novel precision-aiming task (i.e., MOA task) that required participants to move a cursor to an end-goal target in the presence of random moving objects (Mackenzie and Harris, 2017). In addition to demanding a coordinated contribution from feedback and feedforward processes for the control of cursor movement (Wolpert and Kawato, 1998; Elliott et al., 2010), participants had to concurrently monitor the random moving objects in order to avoid a collision and thus the early cessation of the trial. Extending upon MOT, and in a similar way to many tasks performed in daily life and while playing sport, MOA task requires distributed and sustained visual attention across the computer display to continually monitor and guide one’s own movement with respect to the surrounds (Mackenzie and Harris, 2017).

Acquisition of MOA was measured by comparing the effect of practice condition (i.e., treatment effect) on post-test outcome in normal vision. Accordingly, groups practiced MOA in either normal vision or one of two different stroboscopic vision conditions (Nike Vapor Strobe^®^ and PLATO visual occlusion). A control group was included that received no practice. In the stroboscopic vision conditions we used a strobe rate of 1.8 Hz, which was shown with the MOT task to be the most demanding and encouraged greater attentional allocation. We expected that participants practicing in a normal vision condition would acquire the perceptual-motor processes required to satisfy the MOA task, and would thus exhibit better outcome than those that received no practice. Based on the previous findings (Appelbaum et al., 2011, 2012; Clark et al., 2012; Smith and Mitroff, 2012; Mitroff et al., 2013), we anticipated that participants practicing with Nike Vapor Strobe^®^ eyewear would exhibit equivalent or improved learning compared to the normal vision group. Based on the findings from Experiment 1 of the current study, we expected that elimination of vision by the PLATO visual occlusion eyewear would result in the greatest difficulty performing the MOA task, thus limiting adaptation in the processes involved in representing and updating the relevant stimulus information.

### Method

#### Participants

A separate cohort of 52 young adults (*M* = 22.3 years of age, *SD* = 1.4) volunteered to take part in the study. All participants had normal or corrected-to-normal vision and were allocated to one of three experimental groups (normal vision, Vapor Strobe, and PLATO) or a control group (no practice) that were equated according to gender, age, and computer-game playing experience. Participants were excluded from the experiment if they had accumulated 7,500 or more hours playing computer-games. Participants completed informed written consent before taking part in this experiment. The study was reviewed and approved by the research ethics committee of the Research Institute for Sport and Exercise Sciences at Liverpool John Moores University. All procedures were conducted in accordance with the ethical guidelines of Liverpool John Moores University and the 1964 Declaration of Helsinki.

#### Apparatus and Task

The experimental set up consisted of an A3 wide digitizing tablet and stylus (Wacom Intuos3 PTZ-1231W, Saitama, Japan) and a 22-inch CRT computer monitor (Iiyama MA203DT Vision Master 513, Tokyo, Japan), both connected to a desktop computer (HP Compaq 8000 Elite, CA, United States) running Windows XP operating system. The digitizing tablet had a spatial resolution of 5000 dpi, sampling rate of 200 Hz and accuracy of ±0.35 mm, while the monitor operated with a resolution of 1280 × 1024 pixels and a refresh rate of 85 Hz. The monitor and tablet were placed on a desk at a height of 1.0 m. The monitor was located at a distance of 0.9 m from the participant, who was sat on a height-adjustable chair, whereas the tablet was located between the monitor and participant. This arrangement enabled the participant to adopt a comfortable position in which they could clearly see the monitor and easily move the hand-held stylus on the tablet.

**FIGURE 3:**
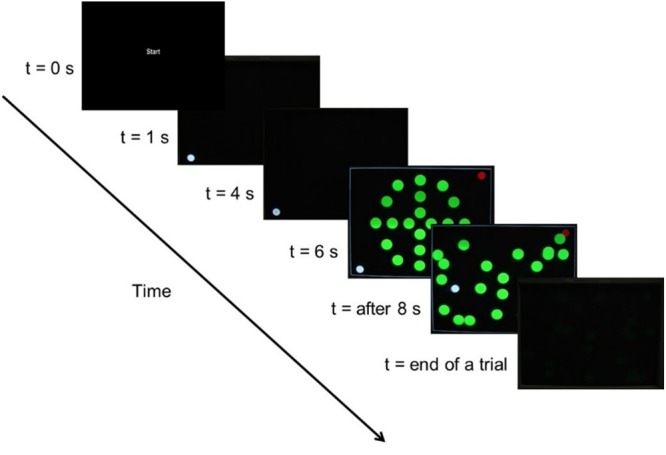
Timeline representation of the MOA task (see text for details).

A MOA task (see **Figure 3**) was created on the host computer that required participants to move a cursor (white circle of 1.4° diameter) to a target (red circle of 1.4° diameter) while avoiding random moving objects (20 green circles of 2.0° diameter). If the white cursor touched one of the green objects, the trial ended and was deemed unsuccessful. If the white cursor reached the red target, the trial ended and was recorded as successful. Stimulus presentation and recording of the hand-held stylus movement was realized using the cogent toolbox implemented in MATLAB (The Mathworks, Inc., MA, United States) on the host computer.

At the beginning of each trial, the word “start” appeared for 1 s. Each trial commenced with the white cursor in either the lower left or right corner of the screen at 1.8° from the vertical and 1.3° from the horizontal screen edge. The start position of the white cursor changed pseudo-randomly from trial-to-trial, but with an equal probability across all trials of being located at lower left or right corner. The white cursor remained stationary at the start position for 3 s, after which a small black dot (diameter of 0.2°) appeared at the center for 2 s. Participants were instructed to focus their attention on the black dot in preparation for the trial to commence. After 2 s, a static image containing the white cursor, red target, and initial position of the green objects was presented for 2 s. The green objects then moved in accord with eight pre-programmed linear patterns (i.e., eight different trials) and participants were free to move the white cursor with the goal of reaching the red target. The green objects moved with a constant speed of 8.9, 8.6, 5.5, or 5.0°/s, which was maintained for each object throughout a trial (i.e., no acceleration). When an object reached the edge of the screen, it rebounded with an angle that was equal to the angle of incidence. The objects did not rebound upon collision with each other and instead continued along their trajectory without any change. Upon collision between the white cursor and a green object or when participants achieved the target successfully, the trial ended and a blank black screen appeared for 100 ms.

#### Procedure

Before the start of the experiment, participants received an illustration of the screen layout (i.e., objects, target, and cursor) and pre-scripted instructions regarding the aim of the task. They were instructed to use the stylus on the digitalizing tablet to move the white cursor on the screen such that it reached the red target whilst avoiding the green objects. They were unaware of either the gain relationship between stylus and white cursor movement or the number of different movement patterns followed by the green objects. They were also informed of which group they had been allocated to and given the opportunity to inspect the stroboscopic eyewear if appropriate.

Each group completed eight trials in a normal vision pre-test and post-test. The order of the eight trials differed in the pre-test compared to post-test, but was the same for all participants. The experimental groups (normal vision, Vapor Strobe, and PLATO) completed a practice phase comprising 12 blocks of these same eight trials. Within each of the 12 blocks, the eight trials were arranged in a pseudo-random order, which was the same for all participants. Participants in these groups were provided with the opportunity of a 60-s break after every four blocks of trials. The control group remained in their seats facing the blank computer screen for 30 min after the pre-test in order to closely replicate the time it took the other groups to perform their practice phase. No augmented feedback such as movement time or end-point error was provided to the participants.

#### Data Analysis

Overall success was quantified as the arcsine percentage of trials in which the cursor reached the red target. Absolute error (AE) was calculated as the two-dimensional difference in position between the center of target and cursor at the end of a trial; in successful trials AE equaled zero. Preparation time (i.e., time between the start of object movement and cursor movement) and movement time (i.e., time between the start of cursor movement and trial end) were calculated from successful and unsuccessful trials (see Supplementary Figure S1 for analysis of successful and unsuccessful trials separately). Overall success and intra-participant means for the measures of motor behavior were calculated for each block of eight trials at pre-test and post-test, as well as during early (trials 1–32), middle (trials 33–64), and late (trials 65–96) practice.

In order to determine if there was a change in task performance across practice, dependent variables were submitted to separate three groups (Vapor Strobe, PLATO, and normal vision) × three practice phases (early, middle, and late) mixed-factor ANOVA. In the event of a significant main or interaction effect, the Holm-Bonferroni method was used to adjust the *p*-value of *post hoc* pairwise comparisons. For the interaction effects, we controlled familywise error rate at α = 0.05 by sequentially comparing phase (i.e., early vs. middle; middle vs. late) for each level of group to give a total of six pairwise comparisons. To quantify the treatment effect of practice, dependent variables measured at post-test were submitted to a 4-group (Vapor Strobe, PLATO, normal vision, and no practice) ANCOVA, with the pre-test measure included as a covariate. This approach has the advantage of minimizing the impact of any initial group differences in performance due to random assignment and takes into account initial within-group variability in performance for our post-test comparisons of interest (Taylor and Innocenti, 1993). The Holm-Bonferroni method was used to adjust the *p*-value for three pairwise group comparisons in which the Vapor Strobe group acted as the reference category.

### Results

#### Performance

**FIGURE 4:**
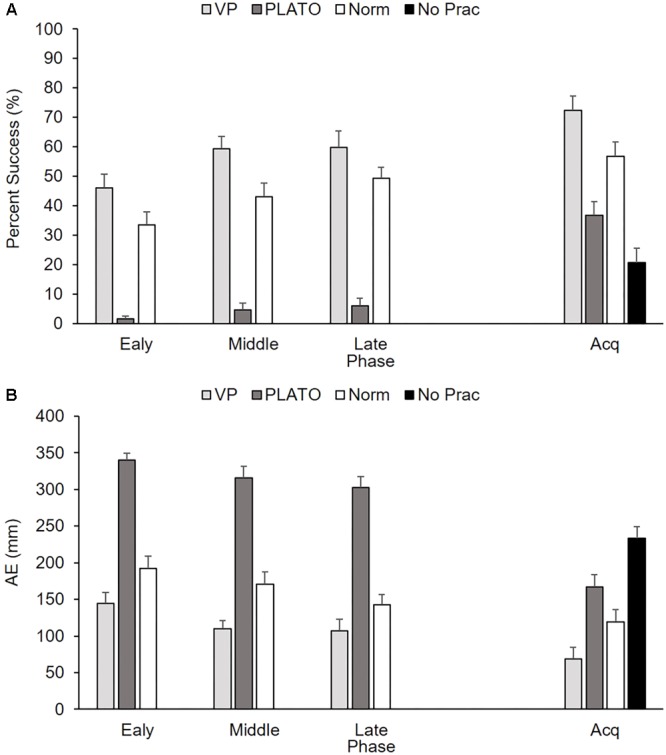
Group mean (+SEM) percent successful responses **(A)** and AE **(B)** as a function of group (VP-Nike Vapor Strobe^®^; PL - PLATO Visual Occlusion; Norm - Normal Vision; No Prac - Control) across practice phase (early, middle, and late) and acquisition (Acq). There is no practice data for the control group as they did not complete any perceptual-motor training. NB. Means for acquisition reflect post-test means adjusted based on the pre-test scores (Success = 20%; AE = 257 mm).

For arcsine percentage of successful trials there was a significant main effect of group, *F*(2,36) = 72.37, *p* < 0.01, $\eta_{p}^{2}$ = 0.80, and practice phase, *F*(2,72) = 12.71, *p* < 0.01, $\eta_{p}^{2}$ = 0.26, as well as a significant interaction between group and practice phase, *F*(4,72) = 7.74, *p* < 0.01, $\eta_{p}^{2}$ = 0.30 (**Figure 4A**). The PLATO group did not improve performance across the three phases of practice (0.05, 0.11, and 0.13), and exhibited a significantly lower percentage of successful trials overall than the Vapor Strobe group (*p* < 0.01). Performance was similar and improved significantly between the early and middle practice for the normal vision (0.56, 0.71) and Vapor Strobe (0.74, 0.90) groups, but not from middle to late practice.

For AE there was a significant main effect of group, *F*(2,36) = 60.72, *p* < 0.01, $\eta_{p}^{2}$ = 0.77 and practice phase, *F*(2,72) = 30.02, *p* < 0.01, $\eta_{p}^{2}$ = 0.46, whereas there was no interaction between group and practice phase, *F*(4,72) = 1.10, *p* > 0.05, $\eta_{p}^{2}$ = 0.06 (**Figure 4B**). Participants in the Vapor Strobe (120.5 mm) group exhibited significantly smaller AE than those in the PLATO (319.5 mm) and normal vision (168.4 mm) groups. All groups exhibited a significant reduction in AE from early to middle, and middle to late practice (225.4, 198.7, and 184.2 mm).

**FIGURE 5:**
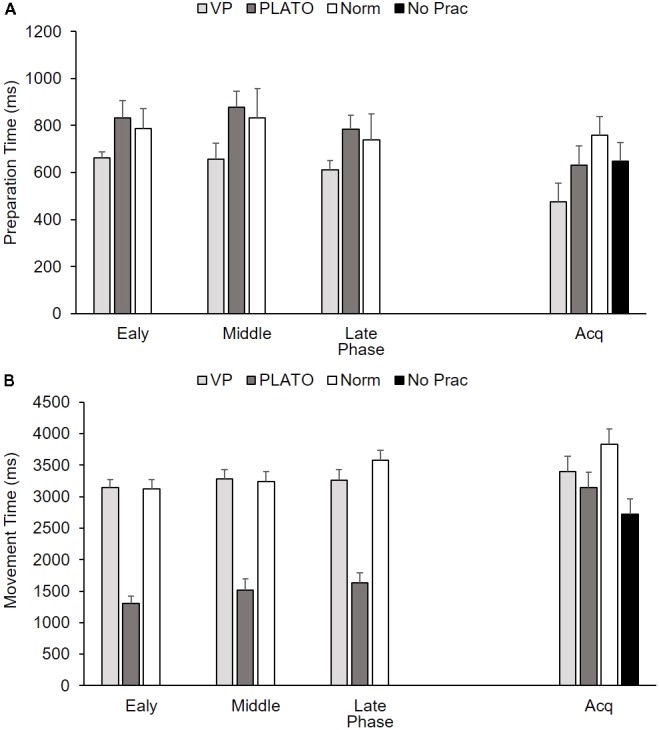
Group mean (+SEM) preparation time **(A)** and movement time **(B)** as a function of group (VP-Nike Vapor Strobe^®^; PL - PLATO Visual Occlusion; Norm - Normal Vision; No Prac - Control) across practice phase (early, middle, and late) and acquisition (Acq). There is no practice data for the control group as they did not complete any perceptual-motor training. NB. Means for acquisition reflect post-test means adjusted based on the pre-test scores (Preparation time = 920 ms; Movement time = 2521 ms).

For preparation time there was a significant main effect of practice phase, *F*(2,72) = 3.97, *p* < 0.05, $\eta_{p}^{2}$ = 0.10, whereas there was no significant main effect of group, *F*(2,36) = 1.79, *p* > 0.05, $\eta_{p}^{2}$ = 0.09, and interaction between group and practice phase, *F*(4,72) = 0.24, *p* > 0.05, $\eta_{p}^{2}$ = 0.01 (**Figure 5A**). While each group exhibited similar preparation time, this was significantly reduced between middle (790 ms) and late (712 ms) practice. For movement time, there was a significant main effect of group, *F*(2,36) = 57.35, *p* < 0.01, $\eta_{p}^{2}$ = 0.76, and practice phase, *F*(2,72) = 12.81, *p* < 0.01, $\eta_{p}^{2}$ = 0.26, whereas there was no interaction between group and practice phase, *F*(4,72) = 1.62, *p* > 0.05, $\eta_{p}^{2}$ = 0.08 (**Figure 5B**). The Vapor Strobe (3230 ms) exhibited a significantly longer movement time than the PLATO group (1480 ms) but not the normal vision (3313 ms) group. All groups significantly increased movement time between the early (2522 ms) and middle (2677 ms) practice (*p* < 0.05), as well as middle and late (2826 ms) practice.

#### Acquisition

There was a significant main effect of group for arcsin percentage of successful trials, *F*(3,47) = 19.35, *p* < 0.01, $\eta_{p}^{2}$ = 0.55, as well as AE, *F*(3,47) = 19.43, *p* < 0.01, $\eta_{p}^{2}$ = 0.55 (**Figures 4A,B**). The Vapor Strobe group exhibited more successful trials and smaller AE than the other three groups. There was no significant main effect of group for preparation time, *F*(3,47) = 2.04, *p* > 0.05, $\eta_{p}^{2}$ = 0.12 (**Figure 5A**), but there was a significant main effect of group for movement time, *F*(3,47) = 3.62, *p* < 0.05, $\eta_{p}^{2}$ = 0.19 (**Figure 5B**). The Vapor Strobe group (3397 ms) exhibited significantly longer movement time than the control group (2719 ms), but not the PLATO (3143 ms) or normal vision (3832 ms) groups.

### Discussion

In this second study, we examined acquisition of a novel precision-aiming task that requires MOA as the participant moves a cursor to a target. Two groups practiced the task in different stroboscopic vision conditions, with a strobe rate (1.8 Hz) that was shown in Experiment 1 to influence the ability to track and disambiguate multiple objects from distractors in a MOT task. Two additional groups were included that either practiced MOA under normal vision or received no practice at all.

Throughout the practice phase participants in the PLATO group showed no improvement in outcome success and consequently remained less successful than those in the Vapor Strobe group. The reduced ability to move the cursor to the final target without being hit by the moving objects was also evident in movement behavior, with the PLATO group exhibiting shorter movement time and greater error on trial cessation than the Vapor Strobe group. Having shown no improvement in task success while practicing in the PLATO group, participants then exhibited worse acquisition (i.e., lower success and higher AE at post-test) than the Vapor Strobe group. Not surprisingly, similarly poor acquisition was exhibited by the control group that received no practice of MOA. Interestingly, however, there was evidence that the Vapor Strobe group exhibited better acquisition (i.e., greater success and lower AE) than those who practiced with normal vision. Consistent with findings from Experiment 1 on MOT, it would appear that participants in the Vapor Strobe group were able to maintain and update the spatio-temporal representation of the cursor relative to multiple moving objects during practice. Importantly, though, in doing so there was enhanced acquisition of the perceptual-motor processes required for success in MOA.

## General Discussion

Motivated by the recent interest in stroboscopic vision training as a means to improve perceptual processing (Appelbaum et al., 2011, 2012; Smith and Mitroff, 2012), and thereby facilitate acquisition of perceptual-motor skill (Mitroff et al., 2013), the current study compared the effect of different stroboscopic vision conditions on MOT and a related precision-aiming task requiring MOA. To this end, we compared two different eyewear that have been commonly used in empirical studies, namely Nike Vapor Strobe^®^ and PLATO visual occlusion spectacles (Translucent Technologies Inc.). The lenses of Nike Vapor Strobe^®^ eyewear switch between more (“open”) or less (“closed”) transparent states, with the latter acting as a neutral density filter that reduced transmission of ambient light in our laboratory setting by 81%. Although not empirically verified with these eyewear, reduced light transmission (i.e., low level light) impacts upon basic function such as visual acuity (von Noorden and Burian, 1959), contrast sensitivity (Owsley et al., 1983), motion perception (Grossman and Blake, 1999), and ocular accommodation (Johnson, 1976). This contrasts with the lenses of the PLATO visual occlusion eyewear that reduced light transmission by only 44% but importantly scattered the light and thus prevented image formation on the retina (Milgram, 1987). For experimental control, we also included conditions in which there was no manipulation of the available visual information (i.e., normal vision) or manipulation was achieved by intermittent presentation of the stimuli on the computer display.

In the first experiment on MOT, we found that participants exhibited a similarly high percentage of successful trials in the Vapor Strobe condition irrespective of strobe rate. A high percentage of success was evident when in the PLATO condition or when viewing an intermittent stimulus presentation but only at the fastest strobe rate. Performance deteriorated significantly with these latter two stroboscopic vision methods for strobe rates less than 3.2 Hz. The different response to these stroboscopic vision methods is consistent with the suggestion that Vapor Strobe eyewear do not eliminate visual motion and form. Indeed, for the MOT task used in the current study, eliminating vision for more than 200 ms impaired participants’ ability to maintain and update the spatio-temporal representation of multiple moving objects between intermittent 100 ms visual samples. Data from a secondary probe reaction task indicated that participants in all groups took longer to react to the random appearance of auditory tones when stroboscopic vision was received at the lowest strobe rate. This is consistent with participants allocating more attentional resource to the primary MOT task when strobe rate was reduced. Importantly, however, increased attention only benefited the MOT task in the Vapor Strobe condition. We suggest that increased attention was necessary for participants to maintain and update the spatio-temporal representation of multiple moving objects when presented with intermittent samples that perturbed normal visual perception of motion and form.

The poor performance exhibited in the PLATO condition or viewing an intermittent stimulus presentation operating at the medium and slow strobe rate may at first seem at odds with previous findings from the MOT task (Scholl and Pylyshyn, 1999). However, vision of multiple moving objects in those studies was eliminated by an occluder (visible or virtual) that was located in a fixed position for the duration of a trial. This resulted in average occlusion durations of 322 ms, which is shorter than those examined here for the lowest strobe rate. Importantly, though, the moving objects in those studies were visible for variable, but long durations, the end of which was predictable because of the fixed location of the occluder. In addition, objects were occluded independently rather than concurrently as in the current study, meaning that there was less demand on visual-spatial working memory to maintain and update the spatio-temporal representation of fewer moving objects (Zelinsky and Todor, 2010). These methodological differences in stimulus presentation between MOT tasks could reasonably account for the lower success found in the current study when vision was intermittently eliminated.

Having determined a strobe rate that influenced performance (i.e., processing and/or outcome) of the MOT task for each stroboscopic vision method, our second experiment examined acquisition of MOA. We found that participants in the PLATO group did not improve outcome success during practice. Indeed, while there was some evidence of a change in aspects of underlying motor behavior, practice performance of the PLATO group generally remained worse than that of the Vapor Strobe group. Moreover, following practice with intermittent elimination of vision for 459 ms, the PLATO group failed to acquire the perceptual-motor processes required for success in MOA when transferred to a normal vision condition. This contrasted with the Vapor Strobe group that exhibited superior acquisition of MOA compared to all other groups, including those who practiced with normal vision. Extending upon previous work (Smith and Mitroff, 2012; Mitroff et al., 2013), these findings demonstrate that acquisition of a perceptual-motor task, which here requires sustained and distributed attention to maintain and update the spatio-temporal representation of participant’s movement relative to multiple moving objects, can be facilitated by practicing in stroboscopic vision that perturbs visual motion and form.

Consistent with previous work on object tracking during occlusion, and the probe reaction findings from our first experiment, we suggest that participants increased attentional resource when faced with the different stroboscopic vision conditions. Flombaum et al. (2008) reported that keeping track of multiple moving objects is an attentionally demanding and effortful task that can draw upon additional attentional resource in challenging situations. According to their so-called high-beams effect, attentional resource is increased during an occlusion for both targets and distractors in order to maintain object persistence and a coherent visual perception. Importantly, these authors also found that attention was increased in the vicinity of an occluder but only when it was occluding a target or distractor. The implication is that attention is not uniformly increased across the display and is instead allocated where needed. In terms of the current study, we suggest that participants increased attention to specific areas of the display during intermittent occlusions in order to facilitate extrapolation of object and cursor trajectories between visual samples. Although not examined here, previous studies have used eye movement recording to indicate the location of overt attention. Indeed, it is known that participants use a gaze strategy that switches between target tracking and centroid tracking in MOT depending on tracking load (Fehd and Seiffert, 2008; Zelinsky and Neider, 2008). It has also been shown that participants selectively shift their gaze during MOT in order to extract relevant information such as an impending collision (Zelinsky and Todor, 2010). Future work on MOA that includes recording of eye movements is required to better understand the overt and covert attentional processes involved in perceptual-motor learning in conditions of stroboscopic vision.

It is well known in the skill acquisition literature that a certain level of attentional load and task difficulty (i.e., challenge point) is required during practice (Guadagnoll and Lee, 2004; Onla-Or and Winstein, 2008; Guadagnoli et al., 2012; Andrieux et al., 2016; Lee et al., 2016). For instance, easy practice can become monotonous due to attentional underload, whereas difficult practice can result in attentional overload (Warm et al., 2008). Neither situation provides the optimal challenge, leading to disengagement and little or no learning. The results of the current study can be interpreted in line with the challenge point hypothesis. For example, while participants who practiced in the PLATO group (1.8 Hz) showed some adaptation in underlying movement behavior, the elimination of vision appeared to be too difficult to facilitate the acquisition of successful MOA. Had we used a faster strobe rate, such as 5.6 Hz (open for 100 ms, closed for 85 ms) that enabled successful MOT in Experiment 1, and achievement of precision-aiming in previous work of (Elliott et al., 1994a,b), we may have provided participants with a more optimal challenge. Indeed, Lyons et al. (1997) found that participants acquired better one-handed catching when practicing with PLATO visual occlusion eyewear operating at a predictable rate of 10 Hz rather than an unpredictable rate that changed between 8, 10, and 14 Hz on a trial-by-trial basis. Still, to our knowledge it has yet to be reported that adaptation to such stroboscopic vision conditions can subsequently benefit behavior when transferred to normal vision. As for participants who practiced in the Vapor Strobe group, it would seem that a strobe rate of 1.8 Hz provided a sufficient challenge to learn the computer-based MOA. This is consistent with our recent finding that participants remained more vigilant when performing coincidence-anticipation in a similar vision condition with a 4 Hz strobe rate (Ballester et al., 2017). Sustained improvements in MOA would likely require a reduction in strobe rate in order to maintain the challenge point and ensure attention remains engaged (i.e., “level-up” procedure; Appelbaum et al., 2011). That said, it is important to recognize that positive effects following training with strobe eyewear do not generalize to all perceptual tasks (Appelbaum et al., 2011) and are not well retained (Smith and Mitroff, 2012). For instance, the “immediate benefit” in accuracy of coincidence-anticipation reported by Smith and Mitroff (2012) following stroboscopic vision training (4 Hz) was no longer present after a 10 min delay. Accordingly, it has been suggested that exposure to stroboscopic vision might be used to enhance performance at key times (e.g., before a baseball player prepares to bat) or to direct attention to particular sources of information (Grooms et al., 2015).

## Conclusion

The visual experience afforded by different stroboscopic vision condition is an important consideration for both perception and perceptual-motor acquisition in tasks requiring sustained and distributed visual attention. Intermittent elimination of visual information for relatively a long duration (i.e., 460 ms) impaired perceptual performance (MOT) and acquisition of a precision-aiming task (MOA). Conversely, use of eyewear with lenses that intermittently reduced light transmission, thereby likely perturbing visual motion and form (for the same duration), did not impair perception and even resulted in superior acquisition of the perceptual-motor task. These findings confirm the potential benefit of practicing lab-based perceptual-motor tasks in stroboscopic vision and indicate that perturbation does not have the same effect as elimination. Further research is required to study the effect of different stroboscopic vision protocols (e.g., strobe rate) and eyewear (e.g., Senaptec Strobe, Visionup, and VIMA Rev Sport), and whether there is positive transfer from such training to perceptual-motor tasks performed in real-world settings.

## Author Contributions

SB, SH, and MU conceived and designed the experiments. MU performed the experiments. SB and MU performed the initial processing of the data, analyzed the data, and wrote the manuscript. All authors contributed to the scientific discussion and reviewed the manuscript. SB coordinated the research.

## Conflict of Interest Statement

The authors declare that the research was conducted in the absence of any commercial or financial relationships that could be construed as a potential conflict of interest.
