# Supplementary figures and images for: Stroboscopic Vision When Interacting With Multiple Moving Objects: Perturbation Is Not the Same as Elimination
Source: Front Psychol. 2018 Jul 25;9:1290. doi: 10.3389/fpsyg.2018.01290 (PMC6068388; doi:10.3389/fpsyg.2018.01290)

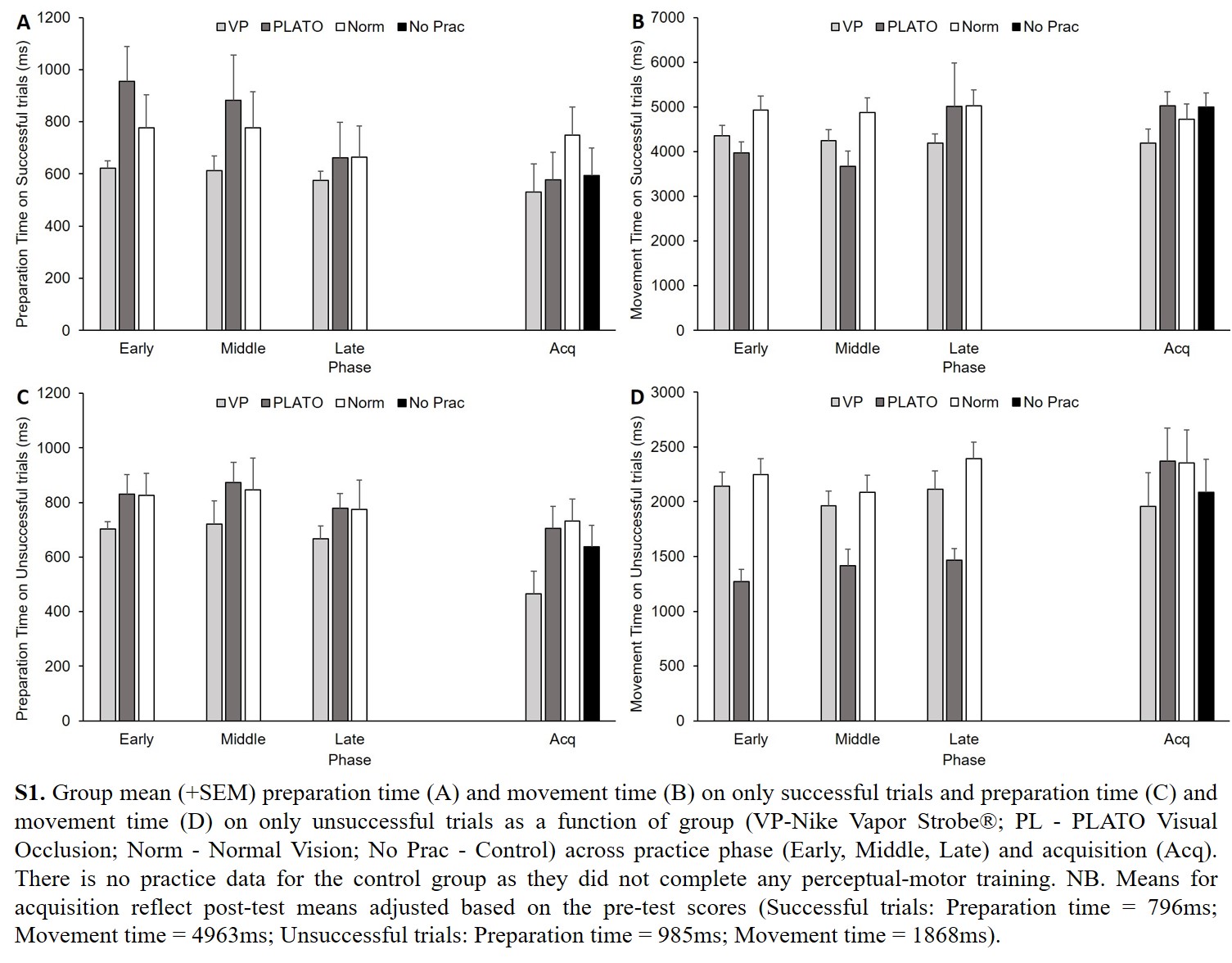

Supplement: Supplementary file 1 [file Image_1.JPEG]
